# Combined role of thromboelastography and coagulation indicators in predicting thromboembolism risk in cancer patients

**DOI:** 10.3389/fmed.2025.1621569

**Published:** 2025-08-26

**Authors:** Liqiu Huang, Wei Dong, Xiaojie Hu, Yu Wang, Lina Zhang

**Affiliations:** ^1^Department of Laboratory, Tangshan Central Hospital, Tangshan, Hebei, China; ^2^Department of Internal Medicine, Tangshan Fengnan District Hospital, Tangshan, Hebei, China; ^3^Department of Emergency, Affiliated Hospital of North China University of Science and Technology, Tangshan, Hebei, China

**Keywords:** thromboelastography, coagulation indicators, thromboembolism, cancer patients, risk

## Abstract

**Objective:**

Exploring the application value of thromboelastography (TEG) and four coagulation indicators in the risk assessment of thromboembolism (TE) in cancer patients.

**Methods:**

Retrospectively analyzed the clinical data of 160 cancer patients. Among them, 45 patients experienced thromboembolism (TE group) and 115 patients did not experience thromboembolism (non-TE group). We analyzed the levels of TEG and coagulation indicators in the two groups of patients and the risk factors for TE in cancer patients.

**Results:**

The Angle and MA of the thromboembolic group were significantly higher than those of the non-TE group, whereas K and R were significantly lower than those of the non-TE group (*P* < 0.05). D-dimer (D-D) and fibrinogen (FIB) levels in the thromboembolic group were significantly higher than those in the non-TE group, while activated partial thromboplastin time (APTT), prothrombin time (PT), and platelet count (PLT) were significantly lower than those in the non-TE group (*P* < 0.05). Binary logistic regression analysis confirmed that angle, maximum amplitude (MA), K, R, D-D, APTT, PT, and PLT were all important influencing factors for the occurrence of TE in cancer patients (*P* < 0.05).

**Conclusions:**

TEG and coagulation index level detection have high application values in the risk assessment of TE in cancer patients.

## Introduction

Cancer has a high incidence worldwide, with rates continuing to rise due to environmental, lifestyle, and genetic factors (1). The increasing incidence of cancer directly correlates with an elevated risk of thromboembolism (TE), which affects around 15% of cancer patients (2). This is particularly concerning given that TE in cancer patients is associated with a significantly higher mortality rate−2–6 times greater than in non-TE patients (3, 4). The elevated incidence of TE further exacerbates the risks of metastasis and adversely affects patient prognosis (5). However, traditional coagulation tests, which provide limited insight into the dynamic nature of blood coagulation, are inadequate for comprehensive risk assessment (6). Therefore, there is a pressing need for more advanced diagnostic methods to evaluate coagulation function in cancer patients. Thromboelastography (TEG) represents a promising tool that allows for a dynamic and comprehensive evaluation of coagulation status, offering a more accurate risk assessment for TE in cancer patients (7).

Coagulation factor testing is a commonly used clinical method, but it can only evaluate partial stages of coagulation, and thus does not provide a complete picture of coagulation status (8). Thromboelastography (TEG) is increasingly recognized as a more comprehensive method for evaluating coagulation function, as it can dynamically measure the stability, strength, and formation rate of blood clots (9). TEG allows for the evaluation of fibrinolytic function, platelet activity, and coagulation factor performance. Various TEG parameters, such as Angle, K, and MA, have been shown to correlate with coagulation abnormalities and thrombotic risk in different clinical settings, such as post-surgical and trauma patients (10). However, its application in cancer patients, especially in the context of malignancy-related thromboembolism, remains underexplored (11). Studies in other clinical areas, such as trauma and surgery, have demonstrated the potential of TEG to provide real-time, dynamic insights into blood coagulation, but its role in cancer patients' thromboembolism risk assessment is a research gap that our study aims to address (12).

Therefore, this study aims to investigate whether the integration of thromboelastography (TEG) and traditional coagulation indices—such as D-dimer and fibrinogen—can enhance the prediction of thromboembolism risk in cancer patients. Addressing this clinical question may help establish a more dynamic and comprehensive approach to hypercoagulability assessment in oncology.

## Materials and methods

### General information

Retrospective selection of records of cancer patients admitted to our hospital between June 2021 and February 2023. Patients with TE will be included in the TE group, whereas those without TE will be included in the non-TE group. Among the enrolled patients, the most commonly used systemic chemotherapy regimens included: (1) platinum-based therapies (e.g., cisplatin or carboplatin), frequently employed for lung, ovarian, and colorectal cancers; (2) taxane-based therapies (e.g., paclitaxel or docetaxel), widely used in breast and gynecologic malignancies; (3) combination regimens such as platinum plus taxane, particularly common in the treatment of non-small cell lung cancer and ovarian cancer. These regimens reflect the standard of care for patients with solid cancer in clinical oncology.

The inclusion criteria were as follows: (1) diagnosis of a malignant tumor through pathological examination, including both local and metastatic adenocarcinomas; (2) systemic chemotherapy treatment during hospitalization or outpatient management; (3) age ≥ 18 years; and (4) complete clinical and thromboelastography data available for analysis.

The exclusion criteria were as follows: (1) individuals with important organ dysfunction, such as kidney and liver, (2) breastfeeding and pregnant women, (3) individuals receiving antiplatelet therapy, (4) individuals who had received low molecular weight heparin (LMWH) or other anticoagulants within 72 h before TEG testing, (5) individuals with congenital coagulation dysfunction, (6) individuals with immune system disorders, and (7) individuals with acquired hematological disorders.

### Detection method

(1) TEG examination: collect 4 ml of fasting venous blood from the patient, and implement anticoagulation treatment with sodium citrate (3.2%); place the specimen in a sterile tube containing kaolin, mix well, and let it stand at room temperature for 5 min; extract 340 μl of mixed whole blood and place it into a regular reaction cup (containing 20 μl of calcium chloride) for testing; According to the relevant operating instructions, test with a TEG detector and record Angle, MA, K, and R. (2) Coagulation index level detection: collect 4 ml of fasting venous blood from the patient, place it in an anticoagulant tube [containing sodium citrate (3.2%), centrifuge (3,000 *r*/min, 15 min, centrifuge radius 10 cm)], take plasma and place it in an EP tube, store it at −80 °C for testing; Perform re dissolution at 37 °C before implementation of testing; using the Japanese SYSMEX CS-5100 fully automatic blood coagulation analyzer to detect D-D, FIB, APTT, and PT; PLT was measured using the Chinese Mindary BC-1800 fully automatic blood cell analyzer.

### Observation indicators

(1) TEG indicator level. (2) Coagulation index levels. (3) Risk factors for TE in cancer patients.

### Statistical method

Data were analyzed using SPSS 26.0, quantitative data were described with ($\bar{\chi }\pm s$), and a *t*-test was used *t*-test describe counting data using frequency and composition ratio (%), using χ^2^ Inspection; the risk factors for TE in cancer patients were evaluated using binary logistic regression, and the predictive value of each indicator was evaluated through ROC curves; *P* < 0.05, indicating a statistically significant difference.

## Results

A total of 160 patients were included in this study. Among them, 87 males and 73 females; there were 45 cases in the thromboembolic group and 115 cases in the non-TE group. There was no significant difference in baseline data between the two groups of patients (*P* > 0.05; Table 1).

**Table 1:** Comparison of general information between two groups.

**General information**	**TE group (*n* = 45)**	**Non-TE group (*n* = 115)**	***t*/*χ^2^***	***P*-value**
**Gender**				
Male	25 (55.56)	62 (53.91)	0.035	0.851
Female	20 (44.44)	53 (46.09)		
Age (year)	59.6 ± 9.23	60.96 ± 9.18	0.839	0.403
BMI (kg/m^2^)	23.87 ± 2.49	23.59 ± 2.31	−0.664	0.508
**Tumor type**				
Breast cancer	11 (24.44)	33 (28.7)	1.303	0.728
Lung cancer	17 (37.78)	45 (39.13)		
Gastric cancer	9 (20)	15 (13.04)		
Colorectal or other	8 (17.78)	22 (19.13)		
**Hypertension**				
No	25 (55.56)	48 (41.74)	2.489	0.115
Yes	20 (44.44)	67 (58.26)		
**Diabetes**				
No	36 (80)	85 (73.91)	0.650	0.420
Yes	9 (20)	30 (26.09)		

TE = thromboembolism.

Among all analyzed parameters, thromboelastography (TEG) indicators showed the most significant differences between groups. Specifically, Angle (66.76 ± 5.68 vs. 64.3 ± 4.47 °, *P* = 0.011), MA (67.16 ± 7.4 vs. 57.97 ± 4.74 mm, *P* < 0.001), *K* (1.41 ± 0.37 vs. 1.96 ± 0.66 min, *P* < 0.001), and *R* (4.76 ± 1.13 vs. 6.57 ± 1.47 min, *P* < 0.001) differed significantly between the TE and non-TE groups (Table 2). These results indicate a hypercoagulable profile among patients with thromboembolism.

**Table 2:** Comparison of TEG indicator levels between two groups.

**Group**	***n***	**Angle (°)**	**MA (mm)**	***K* (min)**	***R* (min)**
TE group	45	66.76 ± 5.68	67.16 ± 7.4	1.41 ± 0.37	4.76 ± 1.13
Non-TE group	115	64.3 ± 4.47	57.97 ± 4.74	1.96 ± 0.66	6.57 ± 1.47
*t*		−2.605	−7.728	6.569	8.328
*P*		0.011	<0.001	<0.001	<0.001

TE = thromboembolism; MA= maximum amplitude.

In addition to the TEG indicators, several conventional coagulation parameters also showed statistically significant differences between the TE and non-TE groups. As shown in Table 3, the TE group exhibited higher levels of D-dimer (294.49 ± 40.77 vs. 231.12 ± 46.23 mg/L, *P* < 0.001) and fibrinogen (4.22 ± 0.99 vs. 3.78 ± 0.74 g/L, *P* = 0.008), and lower values of APTT (27.44 ± 3.73 vs. 31.63 ± 4.06 s, *P* < 0.001) and PT (9.47 ± 2.25 vs. 11.71 ± 1.81 s, *P* < 0.001). In contrast, the platelet count was significantly lower in the TE group compared to the non-TE group (145.82 ± 25.03 vs. 164.35 ± 32.42 × 10^9^/L, *P* < 0.001). These findings further support a hypercoagulable state in cancer patients with thromboembolism.

**Table 3:** Comparison of coagulation index levels between two groups.

**Group**	***n***	**D-D (mg/L)**	**FIB (g/L)**	**APTT (s)**	**PT (s)**	**PLT (×10^9^/L)**
TE group	45	294.49 ± 40.77	4.22 ± 0.99	27.44 ± 3.73	9.47 ± 2.25	145.82 ± 25.03
Non-TE group	115	231.12 ± 46.23	3.78 ± 0.74	31.63 ± 4.06	11.71 ± 1.81	164.35 ± 32.42
*t*		−8.048	−2.727	5.988	6.563	3.858
*P*		<0.001	0.008	<0.001	<0.001	<0.001

TE = thromboembolism; D-D = D-dimer; FIB = fibrinogen; APTT = activated partial thromboplastin time; PT = prothrombin time; PLT = platelet count; s = second.

Perform binary logistic regression analysis with Angle, MA, K, R, D-D, FIB, APTT, PT, PLT as independent variables and TE in cancer patients as dependent variables showed that Angle, MA, K, R, D-D, APTT, PT, and PLT were all important influencing factors for the occurrence of TE in cancer patients (*P* < 0.05; Table 4).

**Table 4:** Analysis of risk factors for TE in cancer patients.

**Variable**	***B***	***S.E*.**	***Wald***	***P-value***	***OR***	***95% CI***	
						**Lower limit**	**Upper limit**
Angle	0.207	0.09	5.239	0.022	1.230	1.030	1.468
MA	0.107	0.049	4.707	0.030	1.113	1.010	1.226
*K*	−2.441	1.096	4.962	0.026	0.087	0.010	0.746
*R*	−0.554	0.257	4.644	0.031	0.575	0.347	0.951
D-D	0.031	0.012	6.383	0.012	1.032	1.007	1.057
FIB	0.457	0.435	1.101	0.294	1.579	0.673	3.705
APTT	−0.291	0.134	4.759	0.029	0.747	0.575	0.971
PT	−0.397	0.188	4.466	0.035	0.672	0.465	0.972
PLT	−0.042	0.018	5.343	0.021	0.959	0.925	0.994

TE = thromboembolism; MA= maximum amplitude; D-D = D-dimer; FIB = fibrinogen; APTT = activated partial thromboplastin time; PT = prothrombin time; PLT = platelet count.

**Figure 1:**
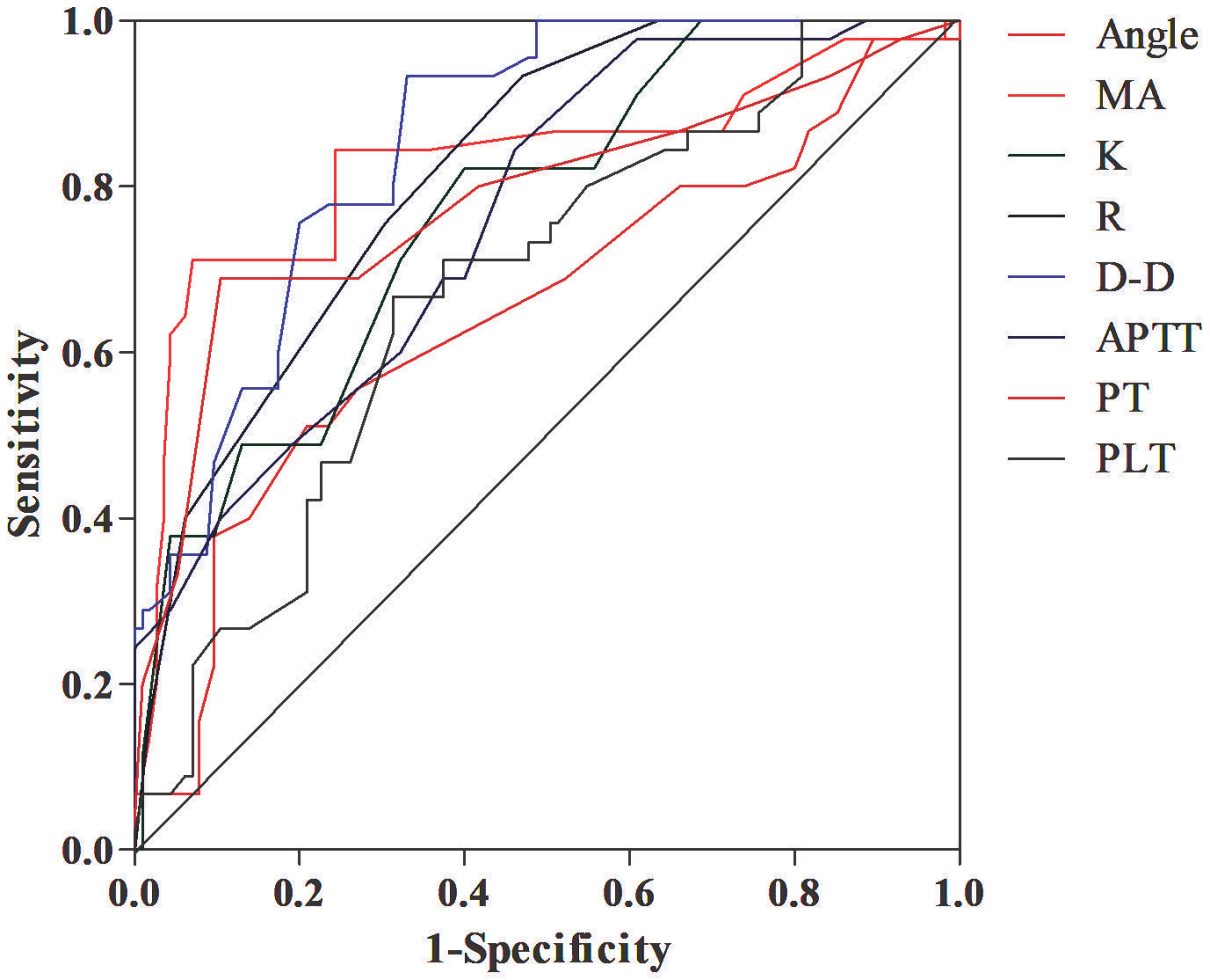
ROC curves of various indicators for predicting TE. MA = maximum amplitude; D-D = D-dimer; FIB = fibrinogen; APTT = activated partial thromboplastin time; PT = prothrombin time; PLT = platelet count.

The ROC curve analysis results showed that Angle, MA, K, R, D-D, APTT, PT, and PLT all have high predictive value for TE, with the predictive value of D-D being the highest (AUC = 0.854; Figure 1, Table 5).

**Table 5:** Evaluation of predictive value of various indicators for TE.

**Variable**	**AUC**	**95% CI**	***P*-value**	**Sensitivity (%)**	**Specificity (%)**	**Cut-off value**
Angle	0.649	0.549–0.750	0.003	51.1	79.1	67.5
MA	0.828	0.745–0.911	<0.001	71.1	93.0	63.5
*K*	0.769	0.692–0.847	<0.001	82.2	60.0	1.7
*R*	0.819	0.753–0.884	<0.001	93.3	53.0	6.5
D-D	0.854	0.796–0.911	<0.001	93.3	67.0	253
APTT	0.761	0.682–0.839	<0.001	84.4	53.9	31.5
PT	0.782	0.693–0.872	<0.001	68.9	89.6	9.5
PLT	0.677	0.588–0.767	0.001	66.7	68.7	146.5

TE = thromboembolism; MA= maximum amplitude; D-D = D-dimer; FIB = fibrinogen; APTT = activated partial thromboplastin time; PT = prothrombin time; PLT = platelet count.

## Discussion

The results of this study showed that Angle, MA, D-D, and FIB in the thromboembolic group were higher than those in the non-TE group, while K, R, APTT, PT, and PLT were lower than those in the non-TE group. Our study identified optimal cut-off values through ROC analysis, such as D-D >253 mg/L and MA >63.5 mm, which slightly differ from those reported in previous studies (13, 14). These variations may be attributed to differences in tumor types, study populations, and the specific TEG instruments used. Thus, we suggest that future research should validate these thresholds in different oncological cohorts to ensure broader applicability. Moreover, our multiple linear regression analysis confirmed that Angle, MA, K, R, D-D, APTT, PT, and PLT were significant predictors of thromboembolism in cancer patients. These findings are consistent with previous clinical studies demonstrating the predictive value of TEG-derived parameters and coagulation indices—particularly Angle, MA, and D-Dimer—in identifying hypercoagulability and TE risk in malignancy (15, 16). This supports the integration of TEG with conventional coagulation testing for improved TE risk stratification. In clinical practice, coagulation function and TEG testing can be performed on cancer patients to assess the risk of TE, and targeted interventions can be administered in a timely manner to reduce the risk of TE and ensure the effectiveness of disease prognosis (17, 18).

Our findings demonstrate that TEG parameters, particularly MA and Angle, along with conventional indicators such as D-Dimer and PT, exhibit significant differences between patients with and without thromboembolism. The combination of TEG and traditional coagulation tests improved the risk assessment of TE in cancer patients. Notably, MA and D-Dimer were independently associated with TE risk, supporting their potential utility as biomarkers for hypercoagulability. This integrated testing approach may facilitate early identification of high-risk individuals and enable timely anticoagulant interventions, thereby improving clinical outcomes. This observation aligns with the known pathophysiology of malignancy-associated thrombosis, where tumor cells can activate coagulation cascades through procoagulant factors, inflammatory cytokines, and endothelial damage (18, 19). TEG, by capturing dynamic clot formation and strength, complements traditional coagulation tests, offering a more holistic view of the prothrombotic tendency in these patients (20).

Several studies have demonstrated the value of coagulation markers and TEG in assessing thromboembolic risk in cancer patients. Gezelius et al. (21) confirmed the predictive value of coagulation indices for venous thromboembolism (VTE) and prognosis in small cell lung cancer. Lobastov et al. (22) reported high sensitivity and specificity of coagulation parameters for predicting postoperative thrombosis in colorectal cancer. However, Lundbech et al. (23) observed perioperative fluctuations in fibrinogen levels without consistent association with TE events, highlighting the need for further validation.

TEG has also been applied in various malignancies as a dynamic tool for coagulation assessment. Feinchtein et al. (24) found that although prostate cancer itself may not be highly thrombotic, TEG abnormalities were observed in ~69% of patients, implicating its role in hypercoagulability evaluation. Similarly, Qin et al. (13) and Lawson et al. (25) confirmed the predictive role of TEG parameters such as R, K, and MA in lung cancer patients, which aligns with the present study's findings.

TEG offers dynamic and holistic insights into the coagulation cascade, complementing traditional tests. Wang et al. (26) showed that TEG outperformed conventional assays in detecting both hypo- and hypercoagulable states, suggesting higher sensitivity and specificity. While routine coagulation testing reflects plasma factor activity at a static point in time, TEG simulates physiological conditions and captures the full process of clot formation and fibrinolysis (25–28).

Furthermore, Gong et al. (14) and subsequent studies have emphasized that TEG's MA parameter—mainly influenced by platelets and fibrinogen—correlates strongly with thrombotic risk. A higher MA indicates enhanced platelet aggregation and stronger clot strength, which is consistent with our findings. This mechanistic evidence supports the utility of TEG in individualized thrombosis risk stratification (14, 29).

To our knowledge, few studies have explored the combined predictive value of thromboelastography and traditional coagulation markers for thromboembolism in patients with malignancies (29, 30). Our findings suggest that this integrated approach may enhance the understanding of coagulation dynamics and provide a promising tool for individualized risk stratification and early intervention in oncology care. Future studies should aim to conduct prospective, multicenter investigations incorporating dynamic monitoring of TEG and coagulation parameters. Additionally, the clinical value of TEG-guided anticoagulation strategies in high-risk cancer patients should be explored. Investigating longitudinal changes in coagulation markers over time and their correlation with the occurrence of thromboembolic events will further elucidate the temporal relationship between coagulation abnormalities and thrombosis risk in malignancy.

### Limitations

First, this was a single-center, retrospective study with a small sample size. Second, only patients with complete clinical data were included and there was a certain selection bias. Third, neither group was randomly assigned, and baseline information may have been imbalanced and biased, which is also one of the shortcomings of our retrospective study. Fourth, TEG may be influenced by human or technical factors. Fifth, the lack of a comprehensive evaluation of other factors on the risk of thrombosis in cancer patients has led to certain biases in the conclusions of this study. Sixth, we did not perform heparinase-modified TEG because patients on recent anticoagulation were excluded from this study, which may limit the applicability of our findings to populations receiving anticoagulation therapy. Finally, future studies with larger, multicenter cohorts are warranted to validate our conclusions.

## Conclusion

TEG and coagulation index level detection have a high application value in the risk assessment of TE in cancer patients. In clinical practice, two comprehensive detection methods can be used to evaluate the risk of TE in patients, thereby guiding the implementation of targeted prevention and treatment in clinical practice.

## Data Availability

The original contributions presented in the study are included in the article/supplementary material, further inquiries can be directed to the corresponding author.
